# Sinapic Acid Mitigates Pentylenetetrazol-induced Acute Seizures By Modulating the NLRP3 Inflammasome and Regulating Calcium/calcineurin Signaling: *In Vivo* and *In Silico* Approaches

**DOI:** 10.1007/s10753-024-02019-0

**Published:** 2024-04-25

**Authors:** Shimaa O. Ali, Heba R. Ghaiad, Ghada F. Elmasry, Noha A. Mehana

**Affiliations:** 1 Department of Biochemistry, Faculty of Pharmacy, Cairo University, Kasr El-Aini Street, Cairo, 11562 Egypt; 2 Department of Pharmaceutical Chemistry, Faculty of Pharmacy, Cairo University, Kasr El-Aini Street, Cairo, 11562 Egypt

**Keywords:** calcium, calcineurin, NLRP3, PTZ, sinapic acid

## Abstract

Sinapic acid (SA) is a naturally occurring carboxylic acid found in citrus fruits and cereals. Recent studies have shown that SA has potential anti-seizure properties due to its anti-inflammatory, antioxidant, and anti-apoptotic effects. The present study investigated the neuroprotective role of SA at two different dosages in a pentylenetetrazol (PTZ)-induced acute seizure model. Mice were divided into six groups: normal control, PTZ, SA (20 mg/kg), SA (20 mg/kg) + PTZ, SA (40 mg/kg), and SA (40 mg/kg) + PTZ. SA was orally administered for 21 days, followed by a convulsive dose of intraperitoneal PTZ (50 mg/kg). Seizures were estimated via the Racine scale, and animals were behaviorally tested using the Y-maze. Brain tissues were used to assess the levels of GABA, glutamate, oxidative stress markers, calcium, calcineurin, (Nod)-like receptor protein-3 (NLRP3), interleukin (IL)-1β, apoptosis-associated speck-like protein (ASC), Bcl-2–associated death protein (Bad) and Bcl-2. Molecular docking of SA using a multistep *in silico* protocol was also performed. The results showed that SA alleviated oxidative stress, restored the GABA/glutamate balance and calcium/calcineurin signaling, downregulated NLRP3 and apoptosis, and improved recognition and ambulatory activity in PTZ-treated mice. *In silico* results also revealed that SA strongly interacts with the target proteins NLRP3 and ASC. Overall, the results suggest that SA is a promising antiseizure agent and that both doses of SA are comparable, with 40 mg/kg SA being superior in normalizing glutathione, calcium and IL-1β, in addition to calcineurin, NLRP3, ASC and Bad.

**Graphical Abstract:**

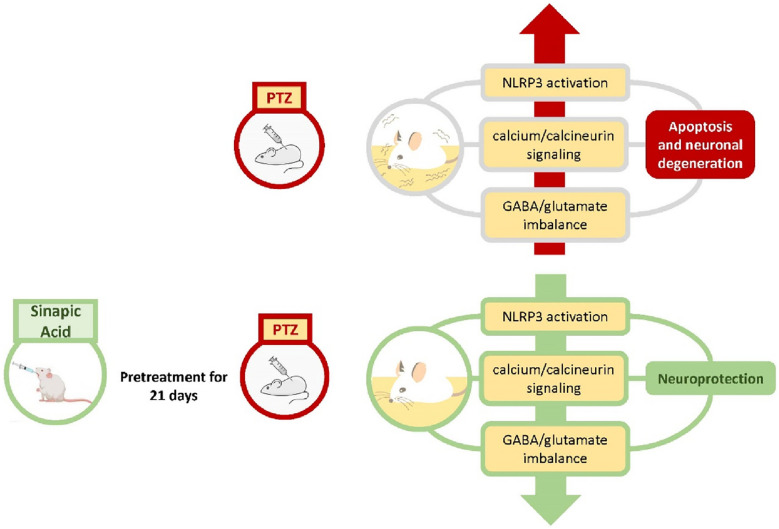

## Introduction

Epilepsy is a serious and deleterious neurodegenerative disease characterized by persistent unprovoked seizures that distress more than 70 million people globally [1]. Seizure episodes are caused by anomalous electrical activity in the central nervous system (CNS) [2]. Despite the availability of more than 30 types of antiepileptic drugs (AEDs), 30–40% of patients develop drug resistance affecting their cognitive function and impairing their psychological behavior with prolonged use [3, 4]. These facts motivated the search for replacement therapy with greater therapeutic potential and fewer side effects.

Various studies have postulated that several etiologies are involved in the pathophysiology of seizures. A disturbed balance between neuronal excitation and inhibition (glutamate/γ-aminobutyric acid (GABA) equilibrium) plays a major role in seizure development [5–7]. Glutamate and GABA transmission can curb the release and/or uptake of other neurotransmitters, affecting neuronal network activity and maintaining normal balance at the cellular level [8].

Additionally, increased calcium influx through glutamate receptors alters excitability [9]. In fact, calcium is a key second messenger in several signaling pathways and has a significant role in the pathogenesis of numerous neurological disorders [10, 11]. Calcineurin is a neuronally abundant, calcium-dependent serine/threonine protein phosphatase that plays an essential role in neuronal excitability as well as apoptosis [12]. Disturbances in cellular calcium disrupt several crucial cellular cascades through calcineurin, including the regulation of neuronal plasticity [13] and the induction of neuronal apoptosis [14]. Accordingly, alterations in cellular calcium levels stimulate calcineurin-mediated signaling, which is intricately involved in acute seizures and status epilepticus [15].

Furthermore, neuroinflammation, the generation of reactive oxygen species (ROS), and oxidative status contribute significantly to epilepsy progression [16]. Interleukin-1β (IL-1β), IL-6, and tumor necrosis factor-α (TNF-α) are the main epileptogenic cytokines, in addition to the transcription factor nuclear factor kappa-light-chain-enhancer of activated B cells (NF-κΒ). Collectively, these factors all have an impact on increased glutamate levels, leading to abnormal neurotransmission and epileptic seizures [17].

Recently, numerous studies have focused on the impact of neuroinflammation on the promotion of seizure development by neuronal excitability [18–20]. Inflammasome (Nod)-like receptor protein-3 (NLRP3) is a cytosolic protein that combines with an adaptor protein known as apoptosis-associated speck-like protein containing a CARD (ASC) stimulating caspase-1 [21]. The NLRP3/ASC/Caspase-1 cascade consequently leads to the activation of pro-IL-1β and pro-IL-18 [22, 23]. NLRP3 activation has been shown to influence the pathophysiology of Parkinson’s disease [24], multiple sclerosis [25]**,** and epilepsy [26, 27]. Additionally, an increasing number of researchers have reported that inhibition of the NLRP3 pathway can ameliorate epileptic seizure episodes and cognitive dysfunction [28–30]; therefore, the NLRP3 pathway could be considered a potential treatment target for epilepsy [31–33].

Epileptic seizures affect the balance between Bcl-2-associated death protein (Bad) and Bcl-2-associated X protein (Bax), which are proapoptotic factors, and the antiapoptotic factor B-cell lymphoma 2 (Bcl-2), which in turn activates caspase-3, inducing proteolytic damage and cell death [34].

Sinapic acid (SA) (4-hydroxy-3,5-dimethoxy cinnamic acid or sinapinic acid) is a recognized carboxylic acid that is a member of the phenylpropanoid family and is naturally present in citrus fruits, vegetables, and cereals [35, 36]. Examples of citrus fruits include lemon and Murcott orange [37]. Berries such as strawberries and cranberries also possess a good quantity of SA [38]. The SA content in rye, wheat, rice, oats and other cereal grains is found to be 8% to 10% of all phenolic acids [39]. Spices are also found to contain larger amounts of SA [40, 41]. Additionally, Brassica vegetables such as broccoli, cabbage, turnip and radish have been shown to contain SA and its derivatives [42].

SA has been shown to have potent antioxidant, antiproliferative and antiapoptotic effects [35, 43–46]. SA exerts its antiapoptotic effects by decreasing Bax and caspase-3 protein expression and increasing Bcl-2 protein expression levels [43]. Furthermore, the neuroprotective effects of SA have been previously reported to be attributed to its antagonistic effects on the GABA-A receptor [47, 48].

The machinery through which SA exerts its anti-inflammatory effects comprises *in vivo* and *in vitro* inhibition of the NLRP3 inflammasome, caspase-1 activation and IL-1β production [49]. SA effectively reduces inflammation by inhibiting malondialdehyde (MDA), TNF-α and myeloperoxidase expressions in an inflammatory colitis model [50] and by decreasing NF-κB expression, inhibiting its downstream inflammatory cascade in an acute doxorubicin-induced cardiotoxicity model [43]. The current study pointed at discovering the potential effects of SA administration at two therapeutic doses on seizure vulnerability, oxidative stress, neuroinflammation and apoptotic events complicated by the pathophysiology of pentylenetetrazol (PTZ)-evoked acute seizures in mice. PTZ is an antagonist of GABA-A receptors. Sequential repeated injections of a subconvulsive dose of PTZ have been used for triggering chemical kindling seizures, whereas a single intraperitoneal (IP) injection at a high dose induces acute, severe seizures. This animal model of acute seizures has been broadly used to study neuronal, behavioral and biochemical alterations after epileptic seizures [51].

## Materials and Methods

### Animals

Three-week-old *Swiss albino* male mice (23 ± 4 g) were purchased from the National Cancer Institute (Cairo, Egypt). Mice were housed in polycarbonate cages in the animal facility of the Faculty of Pharmacy, Cairo University. Before conducting the experiment, the animals were acclimated to the laboratory conditions. Adequate food and water were available to all groups, and the mice were housed in controlled environments with a temperature of 24 ± 0.5 °C, a relative humidity of 50–60%, and a 12 h light‒dark cycle. The research was conducted in compliance with the National Institutes of Health Guide for the Care and Use of Laboratory Animals (US-NIH, Publication # 85–23) and was authorized by the Ethical Committee of Animal Care and Use at the Faculty of Pharmacy, Cairo University (Approval Number: BC 3199). Every possible measure was taken to minimize any discomfort experienced by the animals.

### Chemicals

PTZ was acquired from the USA's Sigma‒Aldrich Co., and SA was acquired from Belgium's Acros Organics. All treatments were dissolved in a saline solution. Every drug solution was made from a scratch on the day of the trial. The remaining chemicals were of the best analytical grade and were obtained from Merck (Germany) or Sigma‒Aldrich Co. (USA).

### Preliminary Study

To determine the optimal dose for the PTZ-induced acute seizure model, six mice were randomly injected intraperitoneally (IP) with PTZ (40, 45, 50, 55, 60, or 65 mg/kg). Our preliminary data showed that PTZ at a single dose of 50 mg/kg caused acute seizures, as evidenced by an increase in the seizure severity score, indicating more than three consecutive tonic‒clonic convulsions. However, higher doses resulted in increased mortality. The animals were monitored for 30 min following injection, and the first generalized tonic–clonic seizures were recorded [52]. An experimenter who was blinded to the animal information conducted the seizure assessments.

### Experimental Protocol

Notably, a blinded technician applied random animal distribution. A total of 48 mice were randomly divided into six groups (8 mice/group) as Group 1 (Normal control group); mice were injected with 1 ml/kg single dose of IP physiological saline, Group 2 (Acute seizure model; PTZ group); mice were injected with single dose of IP PTZ (50 mg/kg) [53], Group 3 (SA (20 mg) group) [54]; animals were given daily doses of oral SA solution (20 mg/kg) for 21 days, Group 4 (SA (20 mg) + PTZ group); mice were given daily oral doses of SA solution (20 mg/kg) for 21 days followed by single dose of IP PTZ (50 mg/kg), Group 5 (SA (40 mg) group) [54]; mice were given daily doses of oral SA solution (40 mg/kg) for 21 days and Group 6 (SA (40 mg) + PTZ group); animals were given daily oral doses of SA solution (40 mg/kg) for 21 days followed by single dose of IP PTZ (50 mg/kg). The PTZ-treated mice were then placed individually in a transparent acrylic plastic box to record their seizure behavior before being euthanized via cervical dislocation. Brain tissue dissected from the animals was further biochemically assessed.

### Behavioral Scoring of Epileptic Seizures

The animals were placed in Plexiglas^®^ cages (40 cm × 40 cm × 30 cm) shortly after PTZ injection, and their convulsive behavior was recorded on video for 30 min. Using Racine's scoring system [55], the behavioral seizures were categorized into five stages: no response at stage 0, facial movements with ear and whisker saccades at stage 1, myoclonic jerks without rearing at stage 2, unilateral or bilateral limb clonus at stage 3, rearing with bilateral forelimb clonus at stage 4, and generalized tonic‒clonic seizures at stage 5.

### Y-maze (Spontaneous Alternation)

The Y-maze spontaneous alternation behavior test assesses the spatial working memory of rodents. The experiment was conducted in a maze with three arms (55 cm × 10 cm × 15 cm) at an angle of 120°. Each mouse was placed in the center of the maze and allowed to explore the three arms for 5 min. Rodents preferred to investigate previously unexplored and novel arms of the maze because of their innate desire to explore the new environment by recalling previously visited arms [56]. Entries in each arm were observed and recorded for 5 min, with successive entries into the three arms on overlapping triplet sets, referred to as alternation. The percentage of spontaneous alternation was calculated using the following formula: Spontaneous alternation percentage (SAP) = [(no. of alternations)/Total arm entries-2)] × 100 [57]. In addition, the number of total arm entries was used as an index of the animal ambulatory activity [58].

### Preparation of Brain Tissue Homogenates

The animal brain tissue samples were mixed with cold phosphate-buffered saline solution and homogenized using a Hermle^®^ Z323 high-speed centrifuge (Germany). The homogenates were centrifuged at 4000 rpm for 10 min at 4 °C. Then, the supernatants were collected and stored at -80 °C until biochemical analysis.

### Biochemical Assessment

#### Determination of GABA and Glutamate Concentrations in Brain Supernatants

The brain GABA content was estimated using a mouse GABA competitive ELISA kit provided by Abcam (USA). Additionally, the level of glutamate in the brain was determined using a mouse glutamate ELISA kit with the double-antibody sandwich technique provided by MyBiosource (USA).

#### Biochemical Analysis of Oxidative Stress Parameters

Reduced glutathione (GSH) and malondialdehyde (MDA) levels in the brain supernatant samples were determined using commercially available colorimetric kits. A GSH kit was obtained from Cayman Chemical, USA. The assay is based on the ability of GSH to reduce 5,5` dithiobis (2-nitrobenzoic acid) (DTNB) forming, a yellow chromogen whose color intensity can be measured at 405 nm and is directly proportional to the GSH concentration [59]. For MDA determination, in an acidic medium, thiobarbituric acid reacts with MDA to form thiobarbituric acid-reactive products, which can be measured at 534 nm [60]. A thiobarbituric acid reactive substances assay kit was obtained from Abnova, Taiwan.

#### Determination of IL-1β Concentrations in Brain Supernatants

A mouse IL-1β solid-phase sandwich ELISA Kit (Thermo Fisher Scientific, USA) was used to detect IL-1β in the brain supernatants according to the manufacturer’s instructions. The optical density was measured at 450 nm with a microplate reader (Epoch BioTek Instruments Inc., USA).

#### Measurement of Total Calcium Content

Calcium levels in the brain supernatants were measured using a colorimetric calcium assay kit (Cayman Chemicals, USA). The assay involves the formation of a chromogenic complex between calcium ions and o-cresol phthalein, which can be measured at an OD of 575 nm. A microplate reader (Epoch BioTek Instruments Inc., USA) was used according to the manufacturer's instructions.

#### Quantitative Real-time Polymerase Chain Reaction (qRT‒PCR)

Total RNA was isolated with an RNeasy Mini Kit (Qiagen, Germany). An Implen NanoPhotometer P-Class 300 (Implen, Germany) was used to measure the quality and concentration of the obtained RNA. A high-capacity cDNA reverse transcription kit (Applied Biosystems, USA) was used to reverse transcribe the extracted total RNA. Gene expression was determined using Rotor Gene Q (Qiagen, Germany) and Rotor Gene SYBR Green PCR kits (Qiagen, Germany). The initial 5 min of enzyme activation at 95 °C was followed by 45 cycles of denaturation at 95 °C for 5 s and another 10 s of annealing/extension at 60 °C in the thermal cycler protocol. The ΔΔCT method was used to calculate the changes in target gene expression, which were then displayed as fold changes (FC = 2^−ΔΔCT^) [61]. The mRNA levels of the target genes were normalized to that of the housekeeping gene glyceraldehyde 3-phosphate dehydrogenase (GAPDH). The primers used herein are listed in Table 1.

**Table 1:** qRT‒PCR Primer Sequences

Genes	Forward sequence	Reverse sequence
NLRP3	CCCTTGGAGACACAGGACTC	GGTGAGGCTGCAGTTGTCTA
ASC	GGGCCATTCTGTTTCTCTC	CGTTCACCCTGGTTTTGT
Bad	GGGAGCAACATTCATCAGCAGG	CGTCCTCGAAAAGGGCTAAGCT
Bcl-2	GTGGATGACTGAGTACCT	CCAGGAGAAATCAAACAGAG
Calcineurin	GGTGGCTGGAGATGTCCGAGC	GGTGGTTCTTTGAATCGGTC
GAPDH	GAGAAACCTGCCAAGTATG	GGAGTTGCTGTTGAAGTC

### *In Silico* Study (Molecular Docking)

We performed molecular docking of SA to predict its plausible binding modes and interactions in the vicinities of NLRP3 and ASC as target proteins. Autodock Vina software was used to perform the docking protocol [62, 63] with a grid box of 25^3^ Å^3^ centered on MCC950 (PDB ID: 7VTP) and α-maltotriose (PDB ID: 6KI0) in the active site of NLRP3 and the ASC-CARD, respectively, which were retrieved from the protein data bank (PDB) by means of Exhaustiveness of 16. The chemical structure of SA was created using Marvin Sketch. Autodock tools were applied to generate the needed PDBQT files since Vina Autodock requires the receptor and ligands to be in PDBQT format.

SA was docked into the prearranged active sites for NLRP3 and ASC. The screening of the compound was completed using PyRx 0.8 [64]. The RMSD was calculated using DockRMSD [65]. Finally, the free Biovia Discovery Studio 2021 visualizer was utilized to visualize the docking poses.

#### Optimization of the Target Enzyme's Active Sites

The PDB was used for obtaining the cryo-electron microscopy and crystal structures of the selected targets. Human NLRP3 bound to the specific inhibitor MCC950 (PDB ID: 7VTP) [66] and the crystal structure of the human ASC-CARD complex bound to α-maltotriose (PDB ID: 6KI0) [67] were selected for the subsequent docking study.

#### Docking Validation

Authentication of the molecular docking procedure was performed by redocking the two cocrystallized ligands, MCC950 and α-maltotriose, in the active sites of NLRP3 and the ASC-CARD, respectively. The performed validation reproduced the same binding patterns and interactions of the cocrystallized ligands, confirming that the docking setup used was appropriate for the proposed docking study. This was illustrated by the perfect alignment between the self-docked poses and the cocrystallized ligands MCC950 and α-maltotriose, with root mean square deviations (RMSDs) of 1.262 and 0.52 Å and docking scores (S) of -9.2 and -9.9 kcal mol^−1^ in the binding sites of NLRP3 and ASC-CARD, respectively.

### Statistical Analysis

The mean ± the standard error of the mean (SEM) was used to express all numerical values, and the SEM is shown by all error bars in the graphs. When comparing more than two groups, one-way analysis of variance (ANOVA) followed by Tukey’s post hoc test were used to determine the statistical significance. When comparing the differences between two groups, unpaired Student’s two-tailed *t* test was used. GraphPad Prism version 8.4.2 (GraphPad^®^ Software, USA) was used for all the statistical comparisons. Statistical significance was set at *P* < 0.05.

## Results

### Effect of SA on the Seizure Severity Score in PTZ-treated Mice

Table 2 shows the effect of SA on the seizure severity score of PTZ-treated mice. Mice treated with PTZ progressively experienced high Racine scale scores (mainly generalized tonic‒clonic seizure characteristic of stage 5 of the Racine Scale). Compared to PTZ treatment, pretreatment with SA protected the animals from seizures in which the seizure scores markedly decreased (from 0 to 2 on the Racine scale). Moreover, both doses of SA decreased the seizure score from 4.5 to 1.5 and 0.8, respectively (p = 0.0001 for SA (20 mg) + PTZ and p < 0.0001 for SA (40 mg) + PTZ). Furthermore, the seizure score did not significantly change between the two studied doses of SA in PTZ-treated mice.

**Table 2:** Effect of SA on the Seizure Severity Score in PTZ-treated Mice

**Treatment**	**Seizure severity score**
**PTZ (50 mg/kg)**	4.5 ± 0.33
**SA (20 mg/kg) + PTZ**	1.5 ± 0.29^b^
**SA (40 mg/kg) + PTZ**	0.8 ± 0.37^b^

The data are presented as the means ± SEM

^b^significantly different from the PTZ group at p < 0.05

*PTZ *pentylenetetrazol;* SA *sinapic acid

### Effect of SA on PTZ-induced Variations in the Y-maze Test

**Fig. 1:**
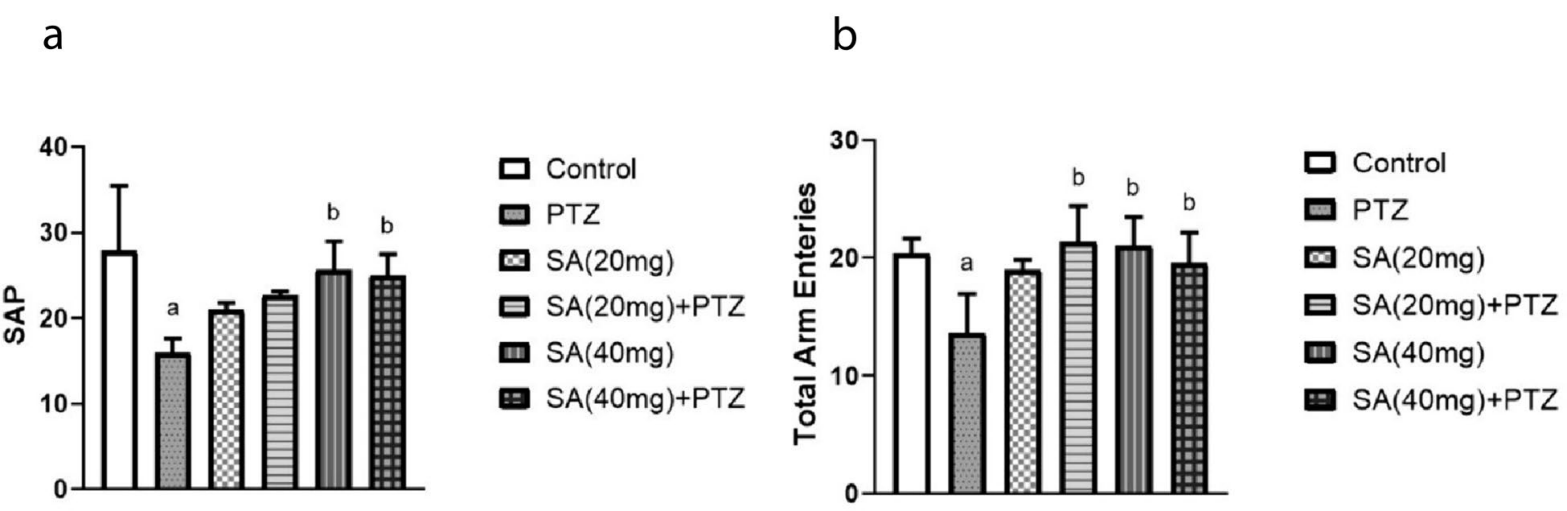
Effect of SA on PTZ-induced variations in the Y-maze test. SAP (**a**) and total arm enteries (**b**). *Each column with a vertical line represents the mean* ± *SEM. *^*a*^* significantly different from the control group, *^*b*^* significantly different from the PTZ group, and *^*c*^* significantly different from the SA (20 mg)* + *PTZ group at p* < *0.05. PTZ, pentylenetetrazol; SA, sinapic acid; SAP, spontaneous alternation percentage.*

The short-term recognition capacity of the animals was assessed using the Y-maze test. Compared with the normal control group, the PTZ-treated group did not recall the instantly visited arms and tended to observe the previsited arms more often, resulting in a 42% decrease in spontaneous alternation (p = 0.0031). In contrast to those in the PTZ group, the animals in the SA pretreatment group exhibited increased spontaneous alternations. Nevertheless, this increase was only significant in the group treated with the higher SA dose, the SA (40 mg) + PTZ group (p = 0.043) (Fig. 1a).

In addition, compared with the normal control group, the PTZ group showed a concomitant significant decrease (p = 0.011) in the number of total entries. Compared to those in the PTZ group, the total number of arm entries in the groups pretreated with both SA (20 mg) and SA (40 mg) significantly increased by 56% and 43%, respectively (p = 0.0034 for SA (20 mg) + PTZ and p = 0.029 for SA (40 mg) + PTZ) in the Y-maze test (Fig. 1b).

### Effect of SA on PTZ-induced Changes in Neurotransmitter Levels

**Fig. 2:**
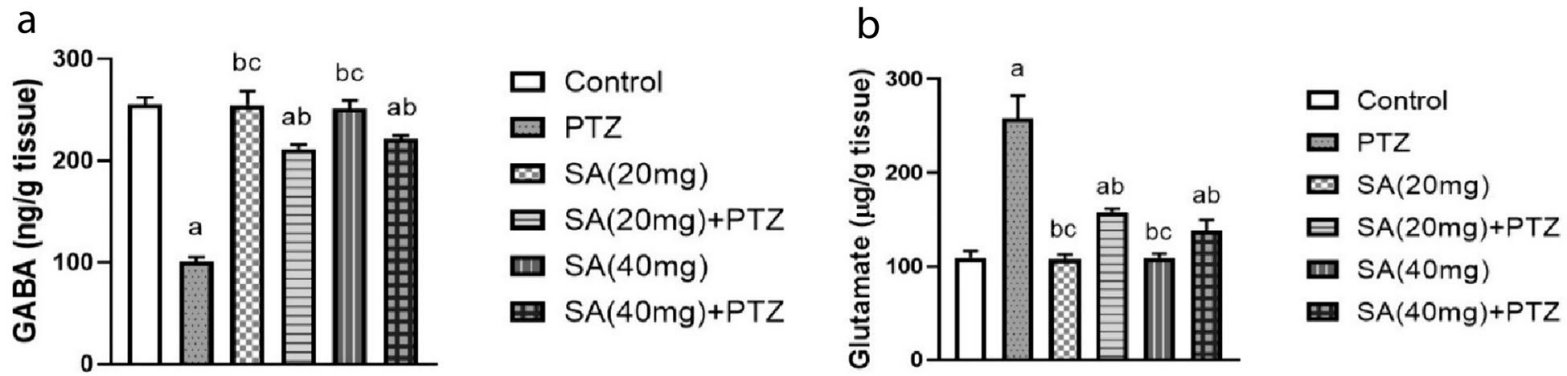
Effect of SA on PTZ-induced changes in GABA (**a**) and glutamate (**b**) neurotransmitter concentrations. *Each column with a vertical line represents the mean* ± *SEM. *^*a*^* significantly different from the control group, *^*b*^* significantly different from the PTZ group, and *^*c*^* significantly different from the SA (20 mg)* + *PTZ group at p* < *0.05. PTZ, pentylenetetrazol; SA, sinapic acid; GABA, γ-aminobutyric acid.*

As shown in Fig. 2a, the administration of PTZ caused a significant decrease in the GABA concentration, which decreased by 61% (p ≤ 0.0001). In contrast, pretreatment with SA significantly amplified the level of GABA by nearly 109% in the SA (20 mg) + PTZ group and by 120% in the SA (40 mg) + PTZ group. Notably, GABA levels were significantly greater in the group pretreated with the higher dose of SA (Student’s *t* test, p = 0.0286). Besides, the glutamate level was significantly greater in the PTZ group than in the normal control group, whereas it was significantly lower in the SA group than in the PTZ group (p ≤ 0.0001 for SA (20 mg) + PTZ and SA (40 mg) + PTZ) (Fig. 2b).

### Effect of SA on Oxidative Stress

**Fig. 3:**
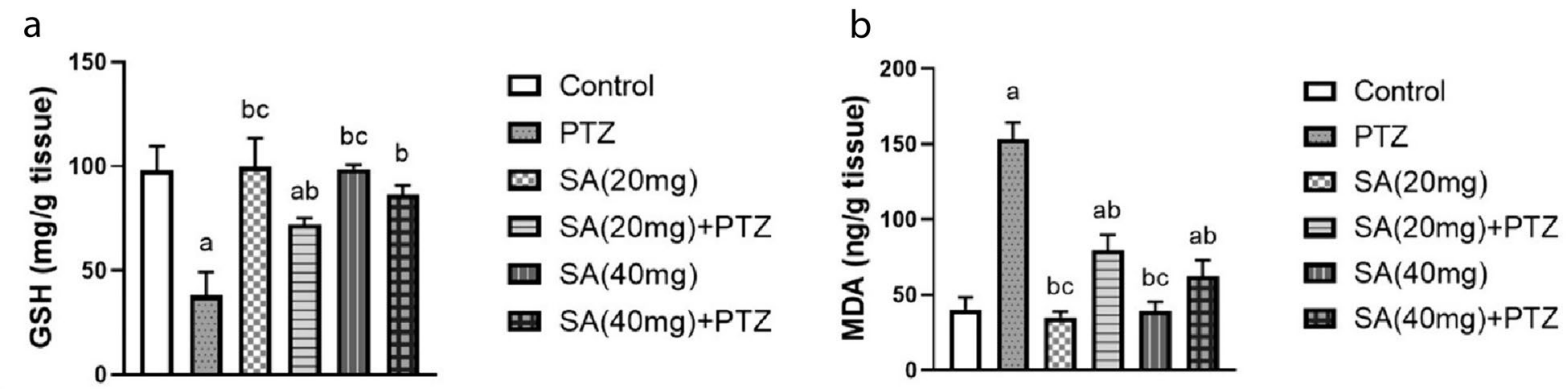
Effect of SA on the oxidative stress parameters. GSH (**a**) and MDA (**b**). *Each column with a vertical line represents the mean* ± *SEM. *^*a*^* significantly different from the control group, *^*b*^* significantly different from the PTZ group, and *^*c*^* significantly different from the SA (20 mg)* + *PTZ group at p* < *0.05. PTZ, pentylenetetrazol; SA, sinapic acid; GSH, glutathione; MDA, malondialdehyde.*

The results of the GSH and MDA measurements in the brain tissue are given in Fig. 3. The administration of PTZ produced a significant 2.5-fold decrease in GSH levels. The level of GSH in both SA groups was significantly greater than that in the PTZ group (p = 0.0005 for SA (20 mg) + PTZ and p ≤ 0.0001 for SA (40 mg) + PTZ) (Fig. 3a)). The findings also showed a significant difference between the two SA doses regarding their effect on brain GSH content (Student’s *t* test, p = 0.0286). On the other side, the MDA level in the PTZ group was significantly greater than that in the normal control group (p ≤ 0.0001). However, pretreatment with SA reversed these changes compared to those in the PTZ group (Fig. 3b).

### Effect of SA on Brain Total Calcium Content and Calcineurin Expression Levels

**Fig. 4:**
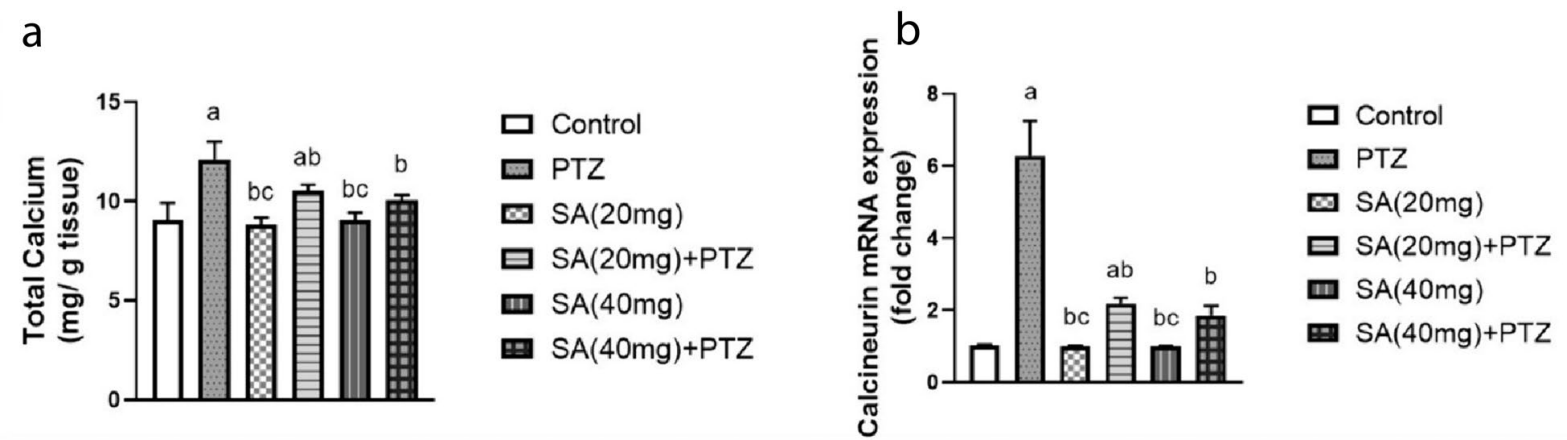
Effect of SA on brain total calcium content (**a**) and calcineurin expression levels (**b**). *Each column with a vertical line represents the mean* ± *SEM. *^*a*^* significantly different from the control group, *^*b*^* significantly different from the PTZ group, and *^*c*^* significantly different from the SA (20 mg)* + *PTZ group at p* < *0.05. PTZ, pentylenetetrazol; SA, sinapic acid.*

As depicted in Fig. 4, mice that received PTZ showed elevated concentrations of total calcium in their brain compared with those in the normal control group (p ≤ 0.0001). Compared with those in the PTZ group, pretreatment with SA significantly decreased the elevated total calcium levels in PTZ-treated mice (p = 0.0114 for SA (20 mg) + PTZ and p = 0.0011 for SA (40 mg) + PTZ) (Fig. 4a). Next, we examined the changes in calcineurin levels and found that PTZ significantly augmented calcineurin expression compared to that in the normal control group. Pretreatment with SA elicited a significant threefold reduction in the calcineurin level compared to that in the PTZ group, with no significant difference among the SA groups (Fig. 4b).

### Effect of SA On the NLRP3 Inflammasome and Its Downstream Effectors in PTZ-treated Mice

**Fig. 5:**
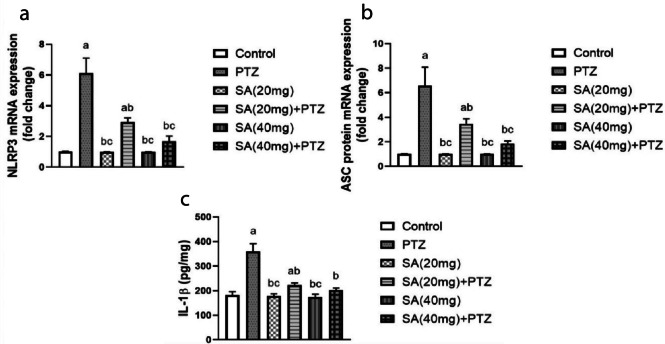
Effect of SA on the NLRP3 inflammasome activation pathway in PTZ-treated mice. NLRP3 (**a**) expression, ASC protein (**b**) expression and IL-1β (**c**) concentration. *Each column with a vertical line represents the mean* ± *SEM. *^*a*^* significantly different from the control group, *^*b*^* significantly different from the PTZ group, and *^*c*^* significantly different from the SA (20 mg)* + *PTZ group at p* < *0.05. PTZ, pentylenetetrazol; SA, sinapic acid; NLRP3, nucleotide oligomerization domain-like receptor protein 3; ASC, apoptosis-associated speck-like protein containing a CARD; IL-1β, interleukin-1beta.*

Compared to those in the normal control group, NLRP3 and ASC gene expression levels were significantly upregulated (sixfold and fivefold increases, respectively) in the PTZ group. However, the expression of these genes was significantly lower in the SA + PTZ group than in the PTZ group, with the higher SA dose (40 mg) having the most potent effect (p = 0.0078 for NLRP3 and p = 0.0232 for ASC) (Fig. 5a, b).

Similarly, PTZ injection significantly boosted the concentration of IL-1β in brain tissue to 197% of that in the normal control group (p ≤ 0.0001) (Fig. 5c). Additionally, pretreatment with SA significantly attenuated the PTZ-induced increase in IL-1β levels. Student’s t test revealed that compared with SA (20 mg) + PTZ-treated mice, SA (40 mg) PTZ-treated mice exhibited significant reductions in NLRP3 and ASC gene expression levels as well as in IL-1β protein concentrations.

### Effect of SA On the Expression Levels of the Apoptosis Markers

**Fig. 6:**
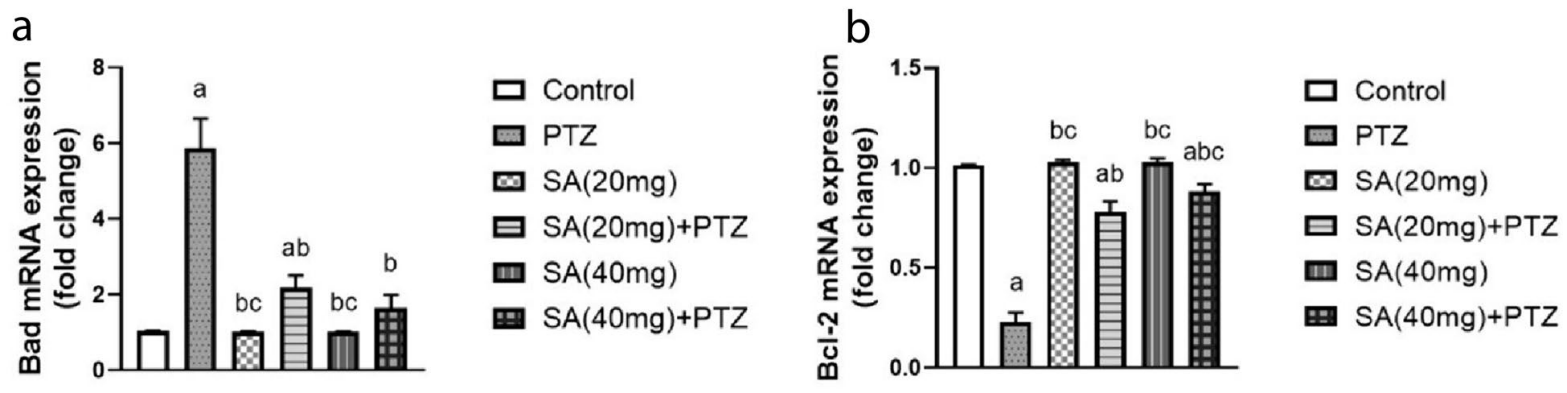
Effect of SA on the expression levels of the apoptosis markers. Bad (**a**) and Bcl-2 (**b**). *Each column with a vertical line represents the mean* ± *SEM. *^*a*^* significantly different from the control group, *^*b*^* significantly different from the PTZ group, and *^*c*^* significantly different from the SA (20 mg)* + *PTZ group at p* < *0.05. PTZ, pentylenetetrazol; SA, sinapic acid; Bad, Bcl-2-associated agonist of cell death; Bcl-2, B-cell lymphoma 2.*

The data in Fig. 6a indicate that Bad expression in the brain was significantly greater in the PTZ group than in the normal control group (p < 0.0001). Nonetheless, pretreatment with both doses of SA significantly decreased the level of Bad compared to that in the PTZ group, as the SA (40 mg) + PTZ-treated group exhibited almost restored normal Bad expression.

In contrast, the expression level of Bcl-2 was significantly lower in the PTZ group than in the normal control group. However, pretreatment with SA markedly enhanced this trend to a level comparable to that of the PTZ group (Fig. 6b). Moreover, pretreatment with a higher dose of SA effectively increased the Bcl-2 expression level more than pretreatment with SA (20 mg) did (p = 0.0039 for SA (40 mg) + PTZ).

### Molecular Docking Simulation Studies

**Fig. 7:**
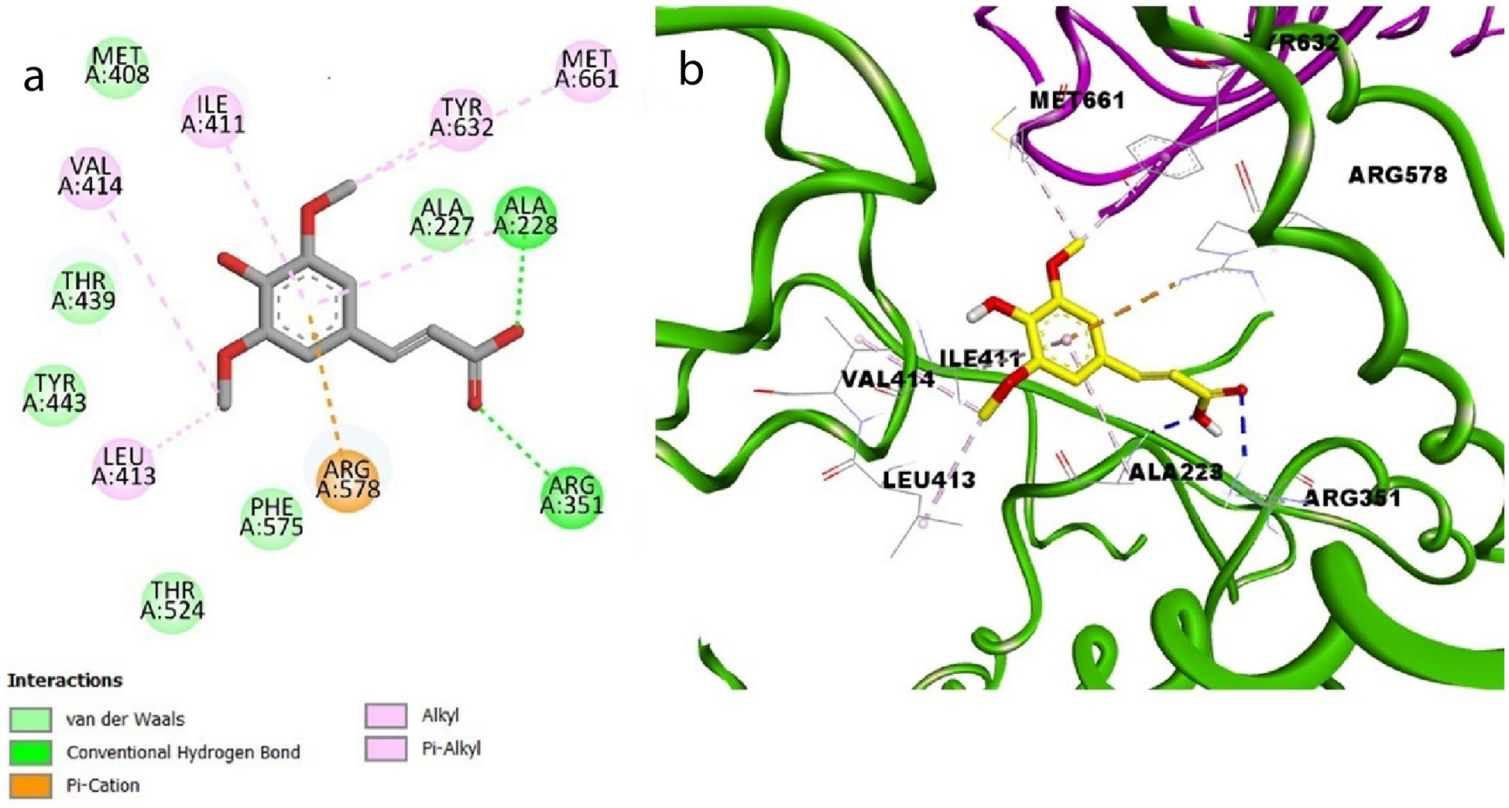
Docking pose of SA in the binding site of NLRP3 in 2D view (**a**) and 3D view (**b**).

SA succeeded in forming a hydrogen bond with Ala228 via its acidic hydroxyl group and another hydrogen bond with Arg351 via its carbonyl moiety in the binding pocket of NLRP3, demonstrating a similar binding pattern to that of the native ligand MCC950, which was engaged in two hydrogen bonds with Arg351 and Arg578 through its carbonyl groups and one hydrogen bond with Ala228 through its NH moiety, in addition to several hydrophobic interactions with different amino acid residues (S1, Fig. 7a).

In addition, π-cation interactions were detected between the phenyl ring of SA and Arg578. Some hydrophobic interactions were also observed with Ile411, Leu413, Val414, Met661, and Tyr632 (S1, Fig. 7a, b). It is noteworthy that SA attained good binding affinity for the target protein NLRP3 (S = -6.0 kcal mol^−1^).

**Fig. 8:**
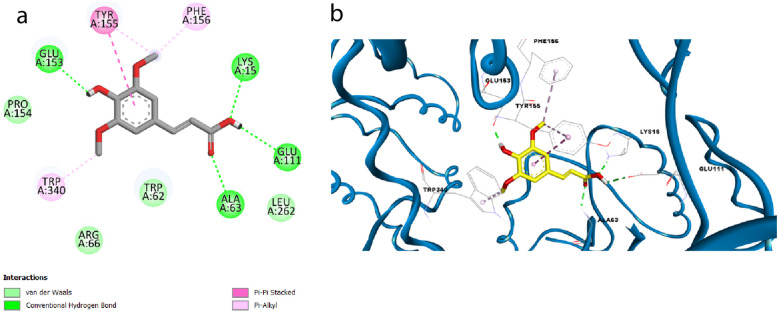
Docking pose of SA in the binding site of ASC in 2D view (**a**) and 3D view (**b**).

Furthermore, SA was also docked into the binding pocket of ASC showing two hydrogen bonding interactions with Lys15 and Glu111 via its carboxylic hydroxyl group and one hydrogen bond with Glu153 via its phenolic hydroxyl group in a similar mode to that of the cocrystallized ligand (α-maltotriose), which formed hydrogen bonds with several amino acid residues*, namely,* Lys15, Trp62, Asp65, Arg66, Glu44, Glu153 and Glu111 (S1, Fig. 7b). Also, SA exhibited an additional hydrogen bond with Ala63 and π-π stacking with Tyr155 through its phenyl moiety and π-alkyl stacking with Tyr155 and Phe156 through its methoxy group (S1, Fig. 8a, b). It is worth mentioning that our tested compound achieved a promising docking score (S = -6.2 kcal mol^−1^).

## Discussion

In the present study, pretreatment with oral SA significantly alleviated oxidative stress, restored the GABA/glutamate neurotransmitter balance, restored calcium/calcineurin signaling and downregulated NLRP3 activation, which collectively ameliorated PTZ-induced seizures and improved spatial working memory in PTZ-treated mice. The biochemical and neurobehavioral findings presented herein reveal, for the first time, a multifaceted neuroprotective mechanism for SA in a PTZ model of acute seizures and highlight a framework for the involvement of three dominant and intermingled axes in seizure pathogenesis related to calcium/calcineurin signaling, NLRP3 activation and the GABA/glutamate balance.

Several compounds, including PTZ, tetanus toxin, penicillin, N-methyl-D, L-aspartate, and strychnine, are utilized to induce acute seizures in animal models. In fact, acute seizure models, especially the PTZ model, have been broadly employed for the evaluation of AEDs [68, 69]. The PTZ-induced convulsive seizure model is the most commonly used model because it is considered a clinical epilepsy model since it mimics epilepsy in people with head trauma [70].

Seizure severity in different experimental seizure models was assessed via behavioral scoring. Racine's scale is an intensity measure frequently applied in acute seizure modeling [10, 71]. According to our behavioral observations, PTZ-treated mice were less sensitive to PTZ after pretreatment with both doses of SA, indicating that SA succeeded in preventing PTZ-induced seizures in mice. As a bioactive phenolic acid, SA and other phenolic acids are widely known for their neuroprotective effects through complex cellular mechanisms [72]. Neuroprotective effects of SA were previously reported in a model of Alzheimer's disease induced by the amyloid β1–42 protein in mice [47] and in a 6-hydroxydopamine-induced hemi-parkinsonian model in rats [48], in addition to oxidative stress-evoked disorders in addition to aging [35, 54, 73].

Changes in brain connectivity and cognitive impairments, in addition to impaired memory and learning functions, are linked to epilepsy in adult patients [74], as are a number of epileptogenesis and acute seizure animal models [75]. The Y-maze test has been used to assess short-term recognition capacity in animals. Spontaneous alternation is a measurement of spatial working memory that can be evaluated by permitting animals to freely discover all arms of the Y-maze depending solely on the innate curiosity of rodents, which directs it to explore formerly unvisited parts. A mouse with integral spatial working memory will remember the previously visited arms and express less drive to visit them again [76].

In our study, PTZ significantly impaired short-term recognition capacity, as indicated by decreased SAP and total arm entries in PTZ-treated mice. PTZ-induced seizures are known to affect short-term memory in rodents [77]. In fact, several earlier studies have associated seizure-induced deterioration in cognitive performance with the extent of neuronal damage, mainly in the hippocampus [78–80]. Herein, SA pretreatment successfully reversed PTZ-induced short-term memory impairments. SA has cerebral protective effects and cognitive-improving effects, as reported previously in a mouse model of scopolamine-induced memory deficit [81]. Additionally, SA was found to be effective in preventing memory loss in intracerebroventricular streptozotocin-induced cognitive dysfunction [82] and in toluene-induced dementia [83].

An ideal working brain requires regulated and well-balanced excitatory and inhibitory input. At the cellular level, glutamate and GABA are the master excitatory and inhibitory neurotransmitters, respectively [84]. Perturbation of the excitatory/inhibitory balance leads to defective signaling, which causes impaired cognitive and motor handling and eventually neuronal damage [8]. In fact, epileptic seizures are known to be associated with an imbalance in GABA/glutamate neurotransmission [84]. PTZ administration causes enhanced glutamatergic transmission with concurrent decreases in GABAergic transmission in the mouse brain [85].

In our study, we established that pretreatment with SA significantly improved GABA levels and decreased glutamate levels in the brain. Interestingly, the high dose of SA (40 mg/kg) succeeded in normalizing the glutamate content in the mice. The previously reported neuroprotective effects of SA have been attributed to the fact that SA is a GABA_A_ receptor agonist. Such GABA_A_ receptor activation is believed to hinder the neurotoxicity induced by agonists of glutamate receptors, such as kainic acid, which is a potent epileptogenic drug [73, 86]. Previously, SA was shown to significantly attenuate kainic acid-induced neuronal apoptosis by activating GABA_A_ receptors and scavenging free radicals [86].

The production of ROS is natural and inevitable, even under normal physiological conditions. In the CNS, oxidative stress underlies the pathogenesis of numerous neurological illnesses, including stroke, neurodegenerative diseases, and epilepsy. In fact, oxidative stress is extensively involved in the initiation and progression of epileptic seizures [87, 88]. PTZ-induced acute seizure modeling has been fundamentally linked to an unbalanced oxidative status [70]. Our results are in accordance with several previous reports in which PTZ-induced oxidative stress was reflected by significantly increased MDA content in brain tissues with concurrent depletion of the antioxidant GSH in the brain [70, 89, 90].

In our study, pretreatment with SA increased the antioxidant GSH and decreased the increase in MDA to ultimately alleviate the oxidative stress status induced by PTZ administration in mice. These results are consistent with the fact that the biological and therapeutic properties of SA are reported to be antioxidative in nature [36, 73, 83, 91].

Excessive stimulation of excitatory receptors results in a calcium burden in the cytoplasm with a consequent production of free radicals, which has been implicated in neuronal death in several neurological pathologies, including seizures [92, 93]. Calcium signaling is a self-motivated second messenger structure that may connect extrinsic signals to complex intracellular activities within neuronal cells. Calcineurin, a heterodimeric calcium-binding serine/threonine phosphatase, comprises a catalytic subunit and a regulatory subunit [10, 15]. Extensive intracellular calcium accumulation and calcineurin upregulation play pivotal roles in neuronal disorders, which has been attributed to the fact that during neurodegeneration and neuronal apoptosis, calcium homeostasis disruptions are mediated by calcineurin [10, 94]. Our findings of increased total calcium content and upregulated calcineurin expression associated with PTZ intoxication are consistent with several previous reports in temporal lobe epilepsy patients [95] and several animal models of seizures [10, 15, 94].

In this study, pretreatment with both doses of SA decreased total calcium levels and downregulated calcineurin gene expression. These results are in line with previous studies in which SA restored calcium levels after ACR-induced neurotoxicity *in vitro* in glioma-derived U87MG cells [73]. Moreover, SA treatment reportedly has calcium-lowering effects in a model of fulminant hepatitis induced by D-galactosamine/lipopolysaccharide in rats [91].

Inflammasome activation plays a central role in epileptogenesis [96]. In animal models of PTZ-induced acute seizures and brain tissues from individuals with pharmacoresistant temporal lobe epilepsy, seizure activity triggers NLRP3 signaling, which can consequently cause apoptosis and neurodegeneration [97–100]. Hence, the use of immunomodulatory drugs that negatively regulate NLRP3 inflammasome activity represents an effective strategy for controlling epileptogenesis and reducing seizures [98].

In our study, the levels of NLRP3 and ASC were significantly decreased by SA pretreatment, which can explain the neuroprotective effect reported herein since SA succeeded in preventing the occurrence of seizures by inhibiting the NLRP3 activation pathway. This postulation can be further proven by the significant decrease in IL-1β reported in our study in both groups pretreated with SA. The capacity of SA to inhibit the activation of the NLRP3 inflammasome was previously reported in rats with diabetic atherosclerosis [101] and in a Kunming mouse model of dextran sodium sulfate-induced ulcerative colitis [102] as well as an intestinal fibrosis model in C57/6BL mice [23].

Another promising finding was that SA formed stable hydrogen bonds with the active site residues of NLRP3 (Ala228 and Arg351) and ASC (Lys15, Glu111, Glu153 and Ala63). Molecular docking analysis was performed for SA in the NLRP3 inflammasome and ASC-CARD to gain deeper insight into the structural features essential for the binding of our ligand to the relevant proteins. Together, the present findings confirmed the *in vivo* data generated from our study that SA treatment significantly decreased the levels of NLRP3 and ASC.

Naturally, the NLRP3 inflammasome is implicated in epileptic neuronal apoptosis. In fact, PTZ is known to induce apoptotic neurodegeneration in animal models of seizures and in HCN-2 neuronal cells [103]. The neuropathologic effects of PTZ can be explained by the ability of PTZ to trigger the neuronal intrinsic apoptotic death program, as indicated by the increased expression of proapoptotic players, including Bad, Bax, caspase-9, caspase-3, and cytochrome-c, and the concurrent decrease in the expression of antiapoptotic Bcl-2 [104–106]. Herein, in our study, the PTZ-treated group exhibited significantly upregulated Bad expression with simultaneous downregulation of Bcl2 expression, which agrees with the findings of the aforementioned studies [104–106].

SA has been found to have antiapoptotic effects on several pathologies, including gentamicin-induced nephrotoxicity in rats [107], cadmium-induced nephrotoxicity [108] and acute doxorubicin-induced cardiotoxicity [43]. Moreover, SA has shown beneficial effects in combating apoptosis in an *in vitro* model of Parkinson’s disease, as SA protected SH-SY5Y human neuroblastoma cells from 6-hydroxydopamine-induced neuronal apoptosis [43].

Additional research is needed to confirm our results and gain a deeper understanding of the role of SA in the regulation of calcium/calcineurin signaling, NLRP3 activation, and other pathways that contribute to epileptic seizures. Moreover, investigating the effect of SA on seizures in female animals could shed light on the potential influence of sex on SA-induced neuroprotection in PTZ-treated animals. Finally, exploring the possible neuroprotective effects of SA in patients with seizures may offer valuable insights for future research.

## Conclusions

In conclusion, our study aimed to provide further insight into the mechanisms of action of SA by investigating its potential binding mode and comparing the effects of pretreatment of epileptic mice with two doses of oral SA, 20 mg/kg and 40 mg/kg. The two doses were comparable in their effects, with some exceptions: the 40 mg/kg SA dose was superior, as it succeeded in normalizing brain GSH, total calcium and IL-1β concentrations, in addition to calcineurin, NLRP3, ASC and Bad gene expression. Moreover, only the higher dose of SA (40 mg/kg) managed to significantly increase SAP in the Y-maze test compared to PTZ-treated animals.

## Data Availability

All data that support the conclusions of the present study are available from the corresponding author upon reasonable request.
